# Current sex and age patterns of rock climbing-related injuries treated in emergency departments

**DOI:** 10.3389/fspor.2025.1555169

**Published:** 2025-07-03

**Authors:** Aimee Madsen, Lydia Pezzullo, Ryan M. Nixon, Kevin R. Vincent, Heather K. Vincent

**Affiliations:** Exercise and Functional Fitness Laboratory, Department of Physical Medicine and Rehabilitation, University of Florida, Gainesville, FL, United States

**Keywords:** rock climbing, rock wall, injury, fracture, sprain, falls

## Abstract

Rock climbing is an increasingly popular sport with >7 million participants in the U.S., with fast growth among youth and women. The purpose of this study was to compare sex- and age-related emergent injury patterns due to participation in rock climbing activity. This was a retrospective study of National Electronic Injury Surveillance System (NEISS) data from climbers who sought ED care (*N* = 1,372; 42.2% female) in U.S. Emergency departments (ED) from 2013 to 2022. The prevalence, type, and anatomical site of climbing injuries by body part and hospital disposition were compared by sex and age bracket [<18 years [pediatric], 18–34 years [adult] and 35–50 years [masters' adult], young] and >50 years [masters' adult, older]) For all climbers, the lower extremity and upper extremity were the most commonly injured sites. Irrespective of sex or age bracket, most injuries occurred in outdoor mountain and rock wall environments. 29.7% were fractures and 19.9% were sprains or strains. 84.6% of patients were treated and released from the ED and 12.3% required admission. For climbers <18 years, 50.0% of injuries were sustained via rock wall, compared to 8.5%–14.7% of other age brackets (*p* < .05). Compared to climbers aged 35–50 and >50 years, the younger two age brackets experienced more sprains/strains. Female climbers had a higher OR for sprains/strains and lower odds for dislocations than males [OR = 1.40 [1.07–1.82] and OR = 0.59 [0.36–0.95], respectively; both *p* < 0.05]. Females and climbers aged <18 years more often experience falls in indoor/rock wall environments with short fall heights (≤3.04 m), whereas more males and climbers aged 18–50 years are injured outdoors from greater heights (3.05–15.2 m; *p* < 0.001). Prevention strategies that can address these vulnerable groups in these environments are warranted to address unique sex and age-related injury diagnoses and fall-related injury risk in the general climbing population.

## Introduction

Rock climbing recently debuted at the 2020 Tokyo Olympic Games (postponed until 2021), reflecting the increasing global popularity of climbing-related activities (1). In 2017, over 7.1 million people participated in one of the four rock climbing disciplines of climbing, which represents significant growth of 4.3 million since 2010 each year (2). Indoor climbing is the fastest growing youth sport in the United States, with a total of 591 new climbing gyms operating in 2021, representing a 400% increase since 2000 (3). The four major disciplines include bouldering (short distance climbing without ropes), top-rope or speed climbing (rope anchoring at the top of the wall, indoor only), sport climbing (ropes anchored at multiple fixed points while ascending) and traditional or trad climbing (temporary rope anchoring placed during the climb) (3). Indoor rock climbing is an increasingly popular sport with a considerable risk of overuse injuries. Despite public interest and participation, medical training in the U.S. does not typically focus on familiarizing physicians with the epidemiology, risk factors, evaluation and treatment of rock climbing-related injuries. Provision of the current status of injuries treated in the ED setting will serve as the foundation for addressing medical preparedness for the general climbing population.

Due to repetitive overload and varying environmental conditions, there are inherent risks for both chronic and acute musculoskeletal injury with different climbing disciplines, irrespective of sex and age. With respect to experience level, evidence differs. Some novice climbers enter the sport with inadequate preparation, or participate in climbing as a recreational activity (particularly with indoor climbing), without climbing experience, which can contribute to injury (4, 5). In contrast, other studies have found that greater climbing experience level may increase injury risk; in youth, competitive climbers are more than twice as likely to be injured than recreational climbers (6) and among adults, injuries are more common among advanced or expert levels (7). Overall, the injury rate for all climbing disciplines ranges between 1 and 4.24 injuries per 1,000 h of participation (8, 9), with unique injury patterns associated with each discipline. For example, traditional alpine and ice climbing are related to elevated risk, with these sports leading to more serious falls from greater heights and greater severity of lower extremity injuries. In contrast, sport climbing is more commonly related to overuse injury to the upper extremity (1). From 2010 to 2020, emergent bouldering injury incidence has increased by approximately 5-fold, and frequently involves injuries to the foot and ankle due to falls; this mixed methods-retrospective and survey study found that females are more likely to sustain falls whereas men are more likely to sustain distortion or overuse injuries (10). Evidence has shown that climber experience and age are either associated with greater injury risk in some studies (9, 11–15) or not associated with injury risk (16–18). Potentially these relationships are modified by climbing discipline and environment. For example, some survey and interview studies show that higher technical difficulty of climbing routes contributes to elevated self-reported injury risk among competitive youth climbers (70% were overuse related, 36% were hand and finger) (8), among adolescents and adults performing intermediate to advanced climbs (18), and to risk for hand and finger chronic injuries among other climber groups (12, 19). Adult indoor climbers self-reported chronic and acute injuries with the sport and the findings revealed that the most common chronic or prolonged injuries occurred at the knee, shoulder, finger, ankle and elbow; only 4% required surgery to the shoulder, upper arm/elbow or ankle (3). Climbers older than 50 years of age had higher rates of overuse injuries (e.g., subacromial impingement syndrome, elbow osteoarthritis), while younger climbers self-reported more acute injuries (e.g., superior labrum anterior and posterior tear). Helmets are not required by any national or international governing body in competition at this time, and as a result, injuries to the face and head can occur. Yoon et al. found in a scoping review of 31 collective retrospective, cross sectional, case series and survey studies that head injuries comprised 0% up to 35% of total injuries (20). Free soloing or ascending without a harness and rope leads to the highest incidence of fatalities (3).

Changes in the sport and the industry of rock climbing include proliferation of indoor rock climbing facilities, improvement in technological advancements, Olympic impact, increased complexity of indoor climbing routes, and interest in “bouldering” by the public (shorter climbs without ropes of harnesses) (5). These changes have produced a broad diversification of the general climber characteristics in the U.S., which has been noted by recent studies to have shifted some injury patterns over time (21). Review of earlier and more recent studies, combined with U.S. national statistics, has revealed that more youth and women have been engaging in various climbing disciplines (1, 22–24), with speculation of a rise in more older climbers to occur over time (17). At present, we do not have a strong understanding of injury patterns, mechanisms, environmental consideration by sex or age group among general climbers. Every year, thousands of climbing-related injuries are treated in emergency departments (ED). One data source that could help clarify the current injury patterns related to emergent rock climbing-related injuries is the National Electronic Injury Surveillance System (NEISS), a database compiled by the U.S. Consumer Product Safety Commission (CPSC) of the federal government. This system collects data on injuries treated in a statistically representative sample of EDs across the United States, enabling researchers to generate “national estimates (NE)”. To produce NE, the data from the sample hospitals were weighted based on factors such as hospital size and geographic location, allowing the data to represent the broader population as such (25). Data from this sample can be used to estimate the total number of similar injuries occurring nationwide, not just within the participating hospitals. To our knowledge, there is very limited comparative research using NEISS to examine rock climbing related injury patterns. One paper has presented general injury patterns with some comparisons by sex and age in 2009 (24), one paper presented head and neck injures alone (26), and one paper included rock climbing among several activities where upper extremity injuries alone were studied (27).

Given that the most recent comprehensive analysis of emergent injuries from NEISS on emergent climbing injuries was published in 2009 (24), it is likely that these results are not representative of the injury trends by sex and age today. Thus, a current understanding of climbing injury patterns by age bracket and sex will be instrumental for medical preparedness surrounding impending competitions and for improving safety mechanisms across climbing facilities. The primary purpose of this study was to compare sex-related injury patterns among the general rock climber population who received treatment in the ED. The secondary purpose was to determine whether injury patterns differed by age bracket [<18 years (pediatric), 18–34 years (adult) and ≥35 years (masters)]. Due to potential differences in risk taking behavior (28) and participation of males in specific types of climbing, it was hypothesized that males would present more frequently for ED visits for lower extremity injuries than females. Also, it was hypothesized that climbers in the age bracket 18–34 years would present to the ED more often for acute injuries.

## 2 Materials and methods

### 2.1 Study design

This was a retrospective epidemiologic study using NEISS. Patients admitted for rock climbing-related injuries to NEISS participating hospitals were extracted from January 1, 2013, to December 31, 2022.

### 2.2 Data source

Approximately 100 hospitals with a 6-bed minimum and a 24-h operating emergency department represented a stratified probability sample from which data were collected. The hospitals were grouped into five strata, where four strata are hospital EDs of different sizes and one represents EDs from children's hospitals. When a hospital is removed from the sampling frame, the highest ranked hospital within the same stratum is invited to replace the hospital that was removed. Weights are recalibrated each year, so that longitudinal analyses of national estimates can occur even with a dynamic sampling frame. On an annual basis, the previous year's data are made available through the CPSC website. Hospital weighs are equal to the inverse of the probability of selection at the stratum level, which are then adjusted for nonresponse or hospital mergers. The total number of hospital ED visits each year is used to generate a ratio adjustment to the weighting of each hospital, based on the anticipated number of hospital visits for the NEISS sample of hospitals. As such, weighs are adjusted annually to mirror the actual number of ED visits in the NEISS sampling frame, which are a known quantity suitable for calibrating the weights (25). Patient information was collected each night from every NEISS hospital for every patient treated in the ED for an injury associated with rock climbing activity consumer products. Each ED is assigned a statistical sample weight based on the inverse of the probability of selection, enabling NEs of injuries to be calculated across the US using the NEISS cases.

### 2.3 Participants

The database was queried for related cases and data were pooled in Excel (Microsoft, Washington USA). A total of 1,397 cases that were tagged as related to “rock climbing” related injuries during this time frame. One member of the study team (HKV) reviewed each case individually to confirm that these were all directly related to participation in rock climbing activity; five cases were found that needed to be removed. Thus, a total of 1,372 cases were found to be appropriate for this analysis.

Inclusion criteria: incurred injury due direct result of participating in the activity of rock climbing, aged 5 years and older, both sexes, all types of climbing activity (indoor and outdoor forms). Exclusion criteria: aged <5 years, injured without direct involvement of rock climbing activity, injuries involved burns, alcohol or drug use. Cases were excluded if the injury was not directly related to rock climbing activity (e.g., climbing on rocks at the beach), or was not sustained directly while participating in the activity (e.g., fell down while camping on a mountain).

Cases were then stratified by sex (male, female) and age bracket [<18 years (pediatric), 18–34 years (adult) and 35–50 years (masters' adult), young and >50 years (masters' adult, older)].

### 2.4 Data extraction and study variables

Variables extracted from the NEISS query included ages, sex, injury location and diagnosis. Clinical narratives associated with each case were reviewed for any additional details on injury mechanism, fall height (if applicable), or other relevant environmental circumstances.

The anatomical locations of injury were downloaded from NEISS as pre-classified codes unique to specific sites in the body. The locations ranged from head to foot and included the head (face, mouth, eye, head, neck), upper extremity (shoulder, elbow, wrist, hand, finger, upper arm, lower arm), lower extremity (hip, upper leg, lower leg, ankle, foot, toe), trunk (upper trunk, lower trunk, pubic region) and “all body parts” (provided as one pre-classified code). For statistical analysis, the injury anatomical locations were comprised of five areas: head, upper extremity, lower extremity, trunk and all body parts.

Injury diagnoses were grouped into six types for statistical analysis. These diagnosis types included: concussion, sprains/strains, soft tissue injuries (inclusive of lacerations, contusions, hematoma, punctures, nerve damage), fracture (stress fracture or acute), joint dislocations, and other (comprised of a mixed of diagnoses such as cellulitis, “swelling” and effusion, “pain”, palpitations, heat illness, compartment syndrome, asthma or shortness of breath, chest pain, cramping, muscle spasms, amputation).

Based on available information in the narrative, the environmental circumstances in which the injury occurred were classified into four general categories. These environments included outdoor (mountain, ice cliff, rock wall or bouldering) and indoor (rock wall, bouldering). If details were not provided, the environment was listed as “not described”. If a fall was listed as the mechanism of injury, fall height was abstracted if available. Fall heights were classified into four brackets from low to high: ≤3.04 m, 3.05–7.62 m, 7.63–15.2 m and ≥15.2 m.

### 2.5 Statistical methods

All analyses were performed using SPSS v. 29 (IBM Corp. Released 2023. IBM SPSS Statistics for Windows, Version 29.0.2.0 Armonk, NY: IBM Corp.). Descriptive statistics were reported as both raw numbers (number of cases) and NEs (calculated using statistical weights provided by the CPSC). Chi-square analysis was used to test whether sex or age bracket differences existed in categorical data (injury location, diagnoses, fall heights). Linear regression analysis was used to determine annual weighted trends in injuries from rock climbing throughout the 10-year study period. The dependent variable was the weighted estimate, and the independent variable was the year. Further, sex and age bracket were individually added to these models of annual injury trends over time. Binary logistic regression was used to clarify the sex differences in location of injury and diagnosis to estimate the odds ratio (OR) and associated 95% confidence interval (CI). The level of statistical significance was established *a priori* at *p* < 0.05.

## 3 Results

### 3.1 Participant characteristics and injury trends

There was a total of 1,372 appropriate cases during the 2013–2022 time frame (NE: 49,737). The mean NE per year was 4,774. The study sample was comprised of 42.2% females and 57.8% males, with corresponding greater NE values for males than females (30,440 vs. 19,297). Table 1 provides the characteristics of the patient pool. Linear regression results indicated that both year and sex were significant contributors to the variance about the NE values (year: R^2^ = .004, F change = 5.613; *p* = .018 and addition of sex to model R^2^ = .006, F change = 8.251; *p* = .004). Age bracket was also a significant contributor to NE (R^2^ = .043, F change = 5.692; *p* < .001).

**Table 1:** Characteristics of the sample population.

Variable	All	Males	Females
Cases (#)	1,372	793	579
Age (year)	26.5 ± 13.4	28.1 ± 13.8	24.4 ± 12.7*
Age bracket (#, %)			
<18 years	339 (24.7)	163 (20.6)	176 (30.4)
18–34 years	756 (55.1)	443 (55.9)	313 (54.1)
35–50 years	183 (13.3)	123 (15.5)	60 (10.3)
>50 years	94 (6.9)	64 (8.0)	30 (5.2)
Race (#, %)			
Asian	87 (6.3)	38 (4.8)	49 (8.5)
African-American	54 (4.0)	26 (3.3)	28 (4.8)
Caucasian	795 (57.9)	466 (58.8)	329 (56.8)
Other/Hispanic	38 (2.8)	24 (3.1)	14 (2.4)
Not specified	398 (29.0)	239 (30.1)	159 (27.5)

Values are raw scores (NEISS cases) and National estimates (NE).

* Denotes different distributions between males and females at *p* < .05.

**Figure 1:**
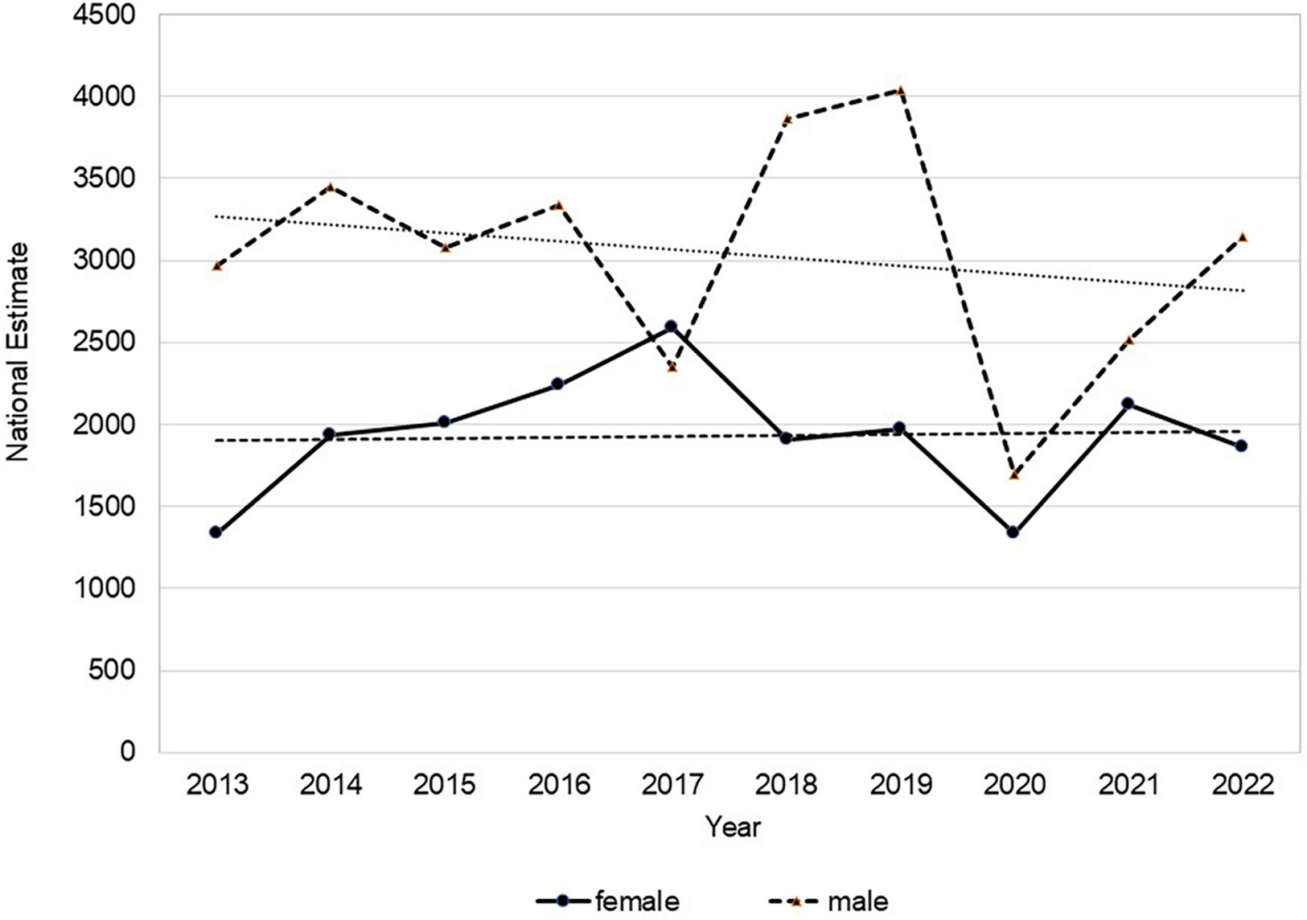
Annual national estimates (NE) of rock climbing-related injuries in males and females who were seen in U.S. emergency departments from the time of 2013 to 2022.

Figure 1 provides these annual NE values for males and females separately. It is notable that the year 2020 had the lowest frequency, with 3,024 injuries reported in US EDs, marking a 49.7% decrease compared to the prepandemic average (2013–2019). In 2021, there were 4,632 injuries, which was an increase of 53.2% from 2020, but this frequency was still below the prepandemic levels by 22.9%. Similarly, the frequency in 2022 was 16.7% lower than prepandemic levels.

### 3.2 Injury location and diagnosis

**Figure 2:**
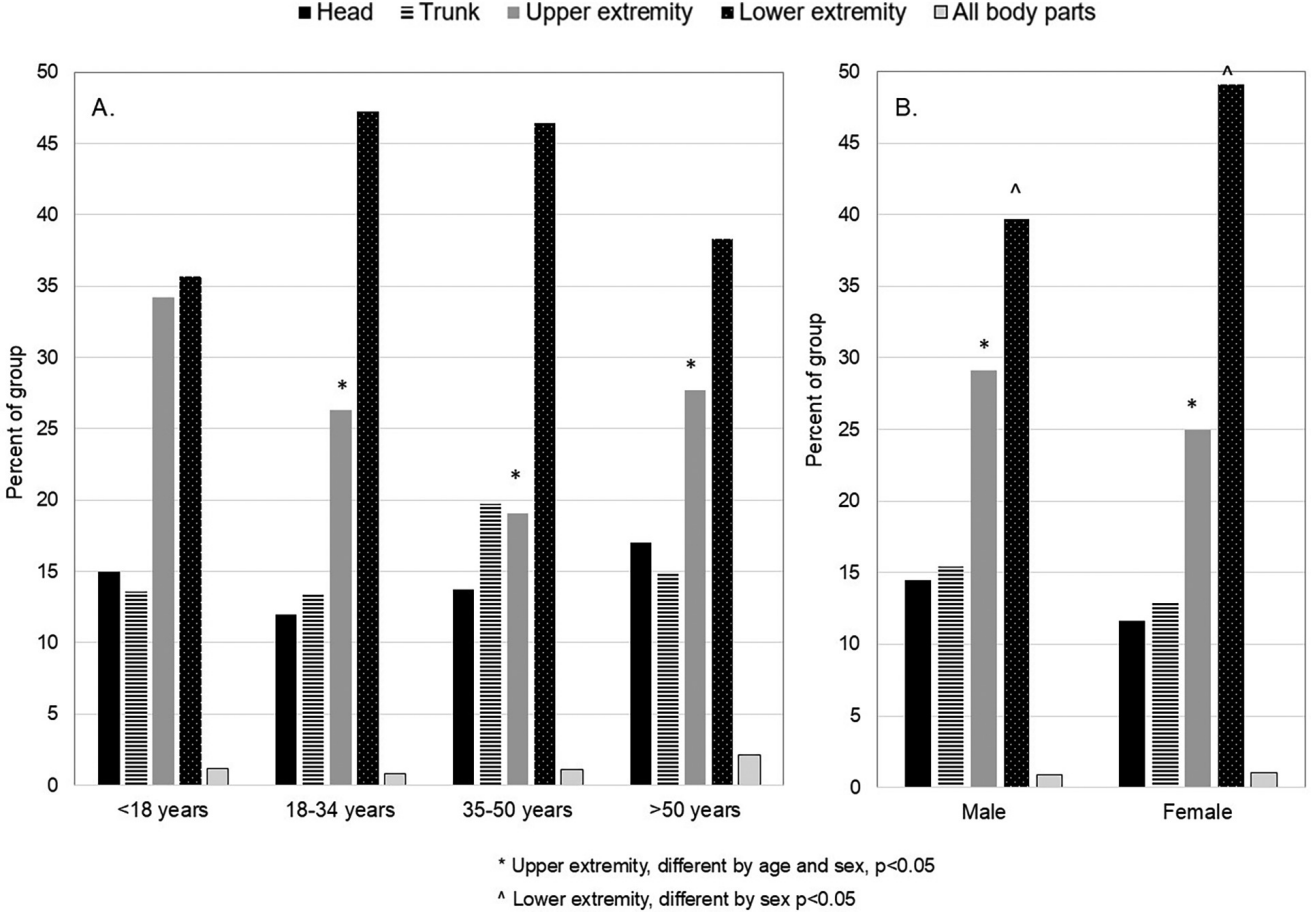
**(A,B)** anatomic location of climbing related injury by age **(A)** and sex **(B)** there was a lower proportion of upper extremity injuries and a higher percentage of lower extremity injuries in the upper three age brackets and among females compared to males.

Figure 2 shows the overall NE values for injury location with percent of the group provided by sex and age bracket. For all patients, the lower and upper extremities were the locations most frequently treated for injury in the ED (NE: 20,958 and 13,834, respectively). More patients <18 years presented with upper extremity injuries than the other age brackets (χ^2^ = 23.291, *p* = 0.03). Females presented to the ED with a higher proportion of lower extremity injuries and fewer upper extremity injuries (χ^2^ = 12.297, *p* = 0.02). Compared to males, females were >50% more likely to incur lower extremity injuries (*p* < 0.05), but less likely to present with upper extremity injury [OR = 0.77 (0.60–0.99); *p* = 0.04]. Females had lower likelihood for head injury [OR = 0.84 (0.62–1.15)], trunk injury [OR = 0.76 (0.54–1.05)] but higher odds for all body parts related injury [OR = 1.22 (0.41–3.70)].

Diagnoses by sex and age bracket are found in Table 2. Age-related differences were detected for the proportion of patients who presented with diagnoses of sprains/strains and dislocation. Specifically, individuals 35–50 and ≥50 years had fewer sprains/strains than the remaining two groups, and those aged 18–34 and 35–50 years had fewer soft tissue related injuries; finally, patients aged 18–34 years presented at least double the proportion of dislocations than the other two age brackets (χ^2^ = 30.975, *p* < 0.01). Compared to males, females had 40% higher odds of incurring sprains/strains (69% of which were in the ankle and knee, and 7% in the shoulder; *p* = 0.01), but 36% lower odds of soft tissue-related injuries and 41% lower odds of dislocations (*p* = 0.03). Among males, 52% of sprains/strains occurred in the ankle and knee, and 11% in the shoulder.

**Table 2:** Injury diagnoses. Odds Ratios (OR ± CI) for females for diagnosis are shown with males as reference.

Injury type	NE Overall	Sex Male (*n* = 793)	Sex Female (*n* = 579)	OR female [± CI]	Age <18 year (*n* = 339)	Age 18–34 year (*n* = 756)	Age 35–50 year (*n* = 183)	Age >50 year (*n* = 94)
Fracture	13,709	223 (28.1)	184 (31.8)	1.21 [0.95–1.52]	99 (29.2)	220 (29.1)	61 (33.3)	28 (29.8)
Sprains/Strains*^,^**	10,151	140 (17.7)	137 (23.7)	1.40 [1.07–1.82]*	75 (22.1)**	161 (21.3)**	29 (15.8)	12 (12.8)
Soft tissue injuries**	11,435	192 (24.2)	98 (16.9)*	0.64 [0.49–0.84]	80 (23.6)	146 (19.3)	36 (19.7)	28 (29.8)**
Dislocations*	3,028	58 (7.3)	26 (4.5)*	0.59 [0.36–0.95]*	11 (3.2)	63 (8.3)	6 (3.3)	4 (4.3)
Concussion	1,387	13 (1.6)	18 (3.1)	1.78 [0.86–3.69]	11 (3.2)	16 (2.1)	3 (1.6)	2 (2.1)
Other**	10,026	167 (21.1)	116 (20.0)	0.97 [0.74–1.27]	63 (18.6)	150 (19.8)	48 (26.2)**	20 (21.3)

Soft tissue injuries include: contusions, lacerations, hematomas, “internal” injuries, nerve damage.

Other injuries include: cellulitis, ’swelling’ and effusion, “pain”, palpitations, heat illness, compartment syndrome, “injury”, asthma or shortness of breath, chest pain, cramping, muscle spasms, amputation.

* Different by sex at *p* < .05.

** Proportions are different by age bracket at *p* < .05.

### 3.3 Environment, injury and falls

Overall, falls were documented in 18.3% (106) of females and in 23.4% (186) of males (*p* = 0.02). Among the four age brackets, falls were reported in 12.6% (43) of <18 year olds, 25.9% (196) of 18–34 year olds, 18.6% (34) of 35–50 year olds and 20.2% (19) of >50 year olds (*p* < 0.001).

Figure 2 provides the proportions of patients who were injured in each climbing environment. There were no statistical age or sex differences in distributions of incurring injury among these environments.

Table 2 provides a comparison of the reported environmental circumstances and heights from which falls occurred. Distributions of fall environment were different for both sex and age bracket; more females and patients <18 years fell during rock wall/indoor climbing compared to respective groups (both *p* < 0.001). More patients who were female and aged <18 years fell from heights ≤3.04 m, whereas males and climbers 18 years and older fell from heights 3.05 m or greater (both *p* < 0.001). The highest proportion of climbers who fell the greatest heights existed among the 35–50 year bracket.

### 3.4 Hospital disposition

Overall, 84.8% of patients were treated and released and 12.2% were admitted. The two fatalities recorded were both male (21 years old, major head injury and skull fracture after 7.63 m fall on a mountain; 30 years old, cardiac arrest while on mountain). Table 3 provides details of disposition after the ED visit. Females were more likely to be treated and released after the ED visit compared to males (88.1% vs. 82.3%; *p* = 0.004). A higher proportion of patients 35–50 years and ≥50 years were admitted to the hospital and were not released compared to younger groups (19.7% and 20.2% vs. 7.7%–11.4%, respectively; *p* < 0.001).

**Table 3:** Disposition after emergency treatment for rock climbing related injuries.

Grouping	Sex		Age			
Disposition	Male (*n* = 793)	Female (*n* = 579)	<18 year (*n* = 339)	18–34 year (*n* = 756)	35–50 year (*n* = 183)	>50 year (*n* = 94)
Treated and released*^,^**	653 (82.3)	510 (88.1)	307 (90.6)	648 (85.7)	142 (77.6)	66 (70.2)
Admitted*	106 (13.4)	61 (10.5)	26 (7.7)	86 (11.4)	36 (19.7)	19 (20.2)
Fatality	2 (0.3)	0 (0.0)	0 (0.0)	2 (0.3)	0 (0.0)	0 (0.0)
Other	32 (4.0)	8 (1.4)	6 (1.8)	20 (2.6)	14 (5.1)	9 (9.6)

* Proportions are different by age bracket at *p* < 0.05.

** Proportions are different by sex at *p* < 0.05.

Patients who were admitted (*n* = 167) had fractures (73.1%), soft tissue injuries (15.6%), dislocation (1.2%), concussion (1.8%), or other (8.4%). The most commonly-reported fracture sites were upper and lower leg (30%), spine and pelvis (27%), ankle (20%) and skull (11%). Males comprised 65% of the fractures admitted to the hospital compared to 35% of females (*p* < 0.001). With respect to age, fractures admitted to the hospital occurred in 17.2% of <18 year olds, 52.4% 18–34 year olds, 20.5% of 35–50 year olds and 9.8% of >50 year olds (*p* < 0.001). The dislocations requiring admission included the ankle and upper spine and both of these were in females.

## 4 Discussion

**Figure 3:**
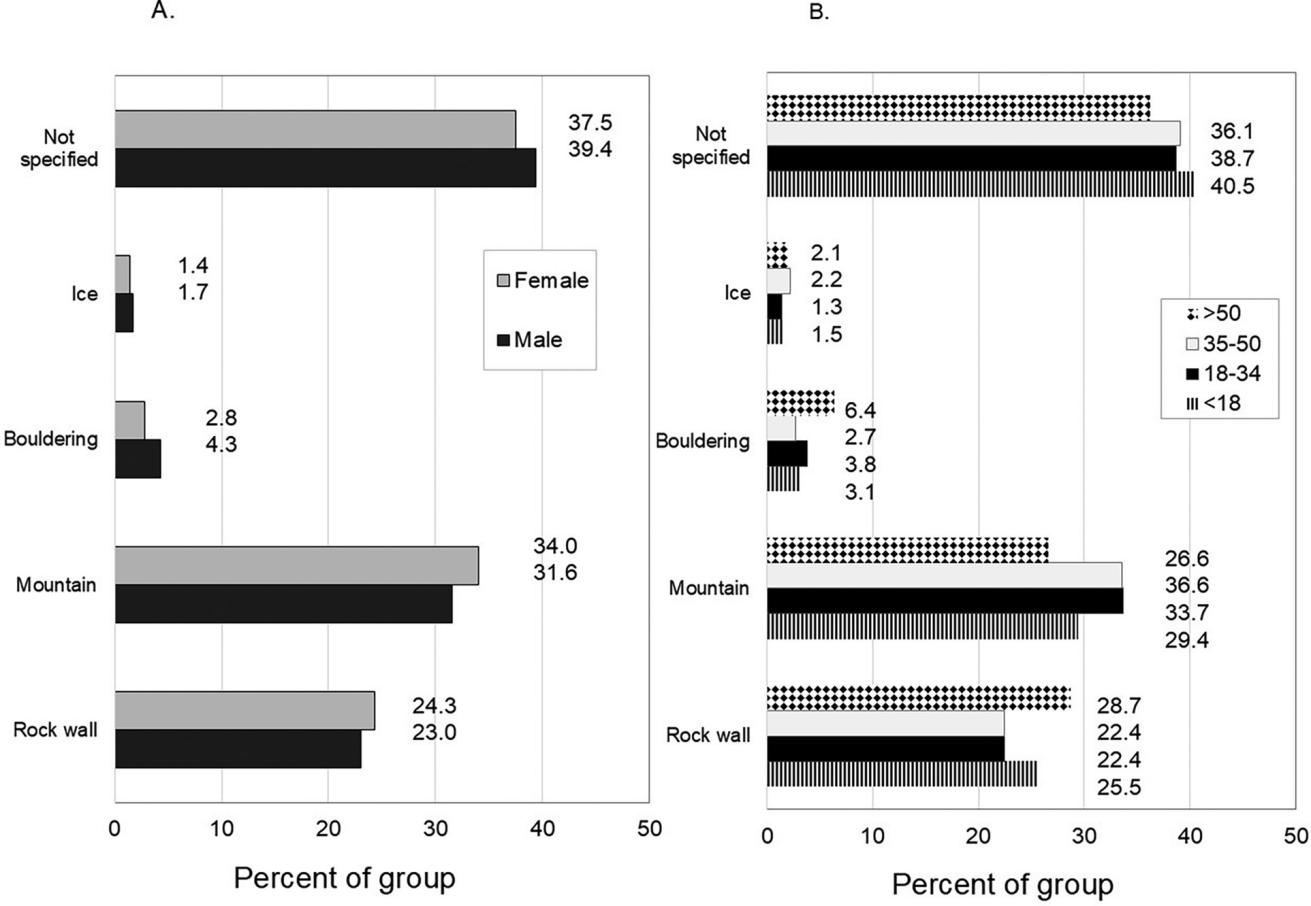
Environments in which rock climbing injuries occurred by sex **(A)** and by age **(B)** values are expressed as a percent of each group.

This study was focused on the comparison of sex and age-related injury patterns among rock climbers who receives treatment in the ED during the years of 2013–2022. Both sex and age were significant contributors to longitudinal injury burden trends as shown by NE values (Figure 1). This analysis provides a current novel view of the emergent acute injury burden among the understudied general climbing population in the U.S. The climber population demographic has shifted over the last decade to include more females and wider age spectrum, with a commensurate rapid expansion of indoor climbing participation even among novices. New findings indicate that the environment on which injuries occur does not differ by sex or age bracket (Figure 3), but the location, diagnosis and incidence of falls do. Females and climbers aged <18 years more often experience falls in indoor/rock wall environments with short fall heights, whereas more males and climbers aged 18–50 years are injured outdoors from moderate heights (Table 4). These patterns reflect unique opportunities to develop age and sex specific preventative efforts to mitigate specific injury types and fall-related injury risk in the general climbing population in the U.S.

**Table 4:** Prevalence, environment and height of falls by sex and age.

Sex				Age				
Fall environment	Male (*n* = 186)	Female (*n* = 106)	*p*	<18 year (*n* = 326)	18–34 year (*n* = 756)	35–50 year (*n* = 183)	>50 year (*n* = 94)	*p*
Rock wall/indoor	34 (18.2)	36 (33.9)	<.001	163 (50.0)	111 (14.7)	18 (9.8)	8 (8.5)	<.001
Mountain/outdoor	79 (42.5)	28 (26.4)		50 (15.3)	247 (32.7)	84 (45.9)	50 (53.2)	
NS	73 (39.3)	42 (39.7)		113 (34.7)	398 (52.6)	81 (44.3)	36 (38.3)	
Fall height (m)	Male (*n* = 186)	Female (*n* = 106)	*p*	<18 year (*n* = 326)	18–34 year (*n* = 756)	35–50 year (*n* = 183)	>50 year (*n* = 94)	*p*
≤3.04 (10)	46 (24.7)	43 (40.6)	<.001	26 (60.5)	54 (27.6)	4 (11.8)	5 (26.3)	<.001
3.05–7.62 (10.1–25)	94 (50.5)	53 (50.0)		14 (32.6)	101 (51.5)	21 (61.8)	11 (57.9)	
7.63–15.2 (25.1–50)	32 (17.2)	5 (4.7)		1 (2.3)	30 (15.3)	4 (11.8)	2 (10.5)	
≥15.2	14 (7.5)	5 (4.7)		2 (4.7)	11 (5.6)	5 (14.7)	1 (5.3)	

NS, environment not specified.

NEISS values, which reflect injury burden in the general U.S. population, were previously 2,238 (24) and 3,816 (1). In the present study, our NE value was at 4,774, showing that the overall climbing-related injury burden in the U.S. has grown in the last decade. When comparing our findings to these earlier NEISS data (1, 24), persistent findings existed. First, the most common injury sites for all climbers remained the lower extremities, followed by upper extremities (Figure 2). Second, sprains/strains, fractures, and soft tissue injuries remained the most common diagnoses. However, we found new sex differences compared to the earlier data. The females in our analysis had 32% greater odds of lower extremity injury and 23% lower odds of upper extremity injury than males. Also, females had a lower risk for dislocations and a 21% greater risk for fractures compared to males (Table 2). The earlier data from Nelson et al. did not detect differences in anatomic location by sex (24), but Buzzacott et al. found that females had greater odds for upper extremity and trunk injury, and lesser odds for lower extremity injury (1). Fracture risk has been shown to be 35%–77% lower among females (1, 24), but dislocation risk is not different for females (24). With respect to age, our findings showed that the climbers ≥18 years had 24%–53% lesser odds of incurring upper extremity injuries than climbers <18 years. Furthermore, the middle two age groups had 49%–54% greater odds of lower extremity injury than climbers <18 years. Diagnoses of sprains/strains were more common among <18 and 18–34 year olds; soft tissue injuries were more prevalent in climbers >50 years. In contrast, Nelson et al. found that 20–39 year olds had a 37% higher odds of sprains/strains than older climbers (24), whereas climbers 40≥ years had 31%–38% lower odds of sprains/strains and 80%–88% greater odds of fractures (1, 24). In our study, fractures of the lower extremity and ankle were the most common reason for hospital admissions, with highest rates in males and patients aged 18–34 years. These evolving injury locations and diagnosis patterns could suggest that females are changing climbing behaviors that include greater risks with falls that injure the lower extremity, particularly in the indoor/rock wall environment.

Other comparative studies characterizing acute climbing injuries often focus on the location, diagnosis, main mechanism of injury and hospital course. Most published evidence has been derived from surveys, databases and prospective survey capture. Some studies focus on specific injury sites such as head/neck (26) or hand/upper extremities (29), or on one age group (8, 17, 30). Data from ED environments and hospital settings that capture a broader injury experience for the age spectrum provide the most relevant direct comparisons for our findings here. In the U.S., nationally representative retrospective studies from other ED databases are limited, however Forrester et al. reported general injury and cost data from 3,275 collective indoor and outdoor “climbing related injuries” collected by the Agency for Healthcare and Quality, Healthcare Cost and Utilization Project's National Emergency Department Sample (31). This study found that the greatest injury type was “multiple body regions” (60%), followed by isolated extremities and head/neck, but did not provide details of mechanism, age or sex breakdown. The relatively low hospitalization cases in this previous work (31), coupled with low incidence of severe injuries could indicate that prevention measures may help mitigate overall injury burden in the general population.

Retrospective studies from hospital/ED settings in Europe and Singapore have shown some variation in injury patterns. Mueller et al. reported that the majority of bouldering injuries from a German population occurred in the lower extremity, with high incidence of sprains (53%) and fractures (22.8%) and dislocations (11.9%) Hospitalizations were necessary in 19.9% of 430 patients (10). Among Swiss outdoor climbers who received care in the ED, the largest proportion involved multiple body regions (38%) or the lower extremities (22%); 68% of the injuries were fractures, with one fatality (32). A retrospective analysis of data from the Austrian Registry of Mountain Accidents performed by Rugg et al. revealed that of the 1,217 acute traumatic injuries, the lower extremity was most affected (38.5%) and 46% of all injuries involved fractures and with a fatality rate of 4.7%. While this Austrian study did not report sex differences in injury patterns, the authors found that: (1) males comprised 78.7% of the cases, and (2) climbers older than 50 years had 4–10 times higher rates of fatalities compared to climbers 18–49 years (33). Falls were the primary mechanism of injury in these investigations. Hospitalizations and fatalities may be more common in outdoor rock, alpine and ice climbing than indoor climbing due to objective environmental challenges such as harsh weather, varied terrain, snow and ice involvement on the surface interface and the need to have additional technical skills for ice axe use and crampons. The collective factors of advanced age and higher risk climbing conditions may have elevated the fatality risk in this last study. Our data did not contain nuances such as hours of climbing participation per year, training volume, complete reporting of climbing discipline or error type when the injury occurred, or volume of lead or traditional climbing. However, a comprehensive survey study found nuances among these factors relating to injury; for example, the likelihood of acute injury changes relative to the type of climbing and hours of exposure (34). The time spent bouldering or lead climbing increased acute injury risk, but decreased relative to volume of traditional climbing (where these climbers tend to be more technically advanced) and chronic injuries may be more likely (34). A small retrospective study from injured climbers admitted to a hospital in Singapore found that most patients had multiple fractures, 72.7% of which were in the lower extremity (35). Three-quarters of the multiple fractures and both open fractures were in females, all of which were sustained from high falls. Irrespective of sex or age, the disciplines of alpine climbing, sport, ice and bouldering have been associated with OR values for 'severe injury' ranging from 2.33 to 4.16 (33). Evidence shows that 83%–92% of patients who sought ED care were treated and discharged (1, 4, 26), which is comparable to our findings (Table 3). The main mechanisms for emergent injury in many studies included falls and being hit/struck by rocks or debris (1, 24, 32). Falls can be associated with more serious injuries and hospital admissions (1, 32). In other studies, patients who go to the hospital and are admitted for care are more likely to have multisite injures or severe isolated extremity injuries (31). Moreover, as many as 20% of these hospitalized injured climbers required skilled nursing or home health services after discharge from the hospital, with 47% reporting a long-term disability (31).

Here, we provide additional details of the environment and fall incidence by fall heights to improve our understanding of injury risk in the general U.S. climber population (Table 4). With fast growth in the U.S. indoor climbing industry, there has been a commensurate increase in youth climbers and diverse participants experiencing climbing. While falls have been reported as a primary mechanism of injury in several other studies (10, 24), we found that falls from lower heights were more common for females and youth in indoor/rock wall environments, whereas males fell more often in mountain/outdoor environments from greater heights. A systematic review indicated that falls are more likely to occur indoors (9), which aligns with our findings. Indoor environments or facilities with rock walls may be more accessible for climbing activity for the general population, but falls in this environment may increase the risk for lower extremity injury. Accidents and falls can happen to any climber, at any height, irrespective of age. To help prepare younger and female climbers against injuries from lower fall heights as we found here, using fall mats (36) consistently and learning how to fall may reduce injury frequency or severity. While additional research is needed, Woollings et al. (9) in their systematic review identified that among the methods used to mitigate injury, only wrist taping and strength training emerged as measures that decreased overall injury risk.

### 4.1 Limitations

There are several limitations that deserve mention. First, due to the nature of the NEISS database, results may produce different results depending on the date ranges used for review and the criteria established to study a specific question. Moreover, the dataset spans recreational climbing across a spectrum, ranging from relatively low-risk indoor rock climbing to higher risk activities like technical mountaineering (31). The level of supervision or support was not captured by this registry and we are not able to comment on the potential safety effects on this point relative to the type of injuries incurred by sex or age bracket. Thus, the complexity of injury risk in the sport of climbing may not fully be represented in the dataset. Second, the information captured in NEISS records is biased toward reporting error. Consistent details, including discipline of climbing, season, consistency of reporting in where exactly the accident occurred, climbing discipline, mechanism if injury, or if other individuals were involved may be missing (37). As such, there is the possibility that statistical findings may be different if all cases reported every detail, and our results should be interpreted with caution. While nonfatal ED data provide insight into rock climbing injury incidence, additional data elements are needed to fully understand the broader burden of injury in this population (38). Third, the methods underlying NEISS data capture include the documentation of the primary location of injury rather than every body part that may have been affected. For example, a patient case that may have been treated in the ED for an acute knee sprain was documented, but did not also document any pre-existing chronic injuries such as finger or shoulder. There is likely underreporting of chronically debilitating injuries in these findings that could alter the OR values for anatomical site or diagnoses. Fourth, although the data from the hospitals included in the analysis are used to estimate the national incidence of injury, accurate regional and state level estimates cannot be made. This means that for many states, only a single hospital within the state contributes to NEISS, and several states do not have any participating hospitals. NE and state populations are used to extrapolate state-specific injury estimates, but these may not accurately represent the real injury burden for the states (37). Not all cases reported fall height when a fall occurred. As such, the statistical findings are a result of available data and may not represent the climbing population as a whole. Fifth, the NEISS dataset does not differentiate whether a climber sought ED care more than once over the 10-year time period we selected here (39). If one climber reported to the ED multiple times, this could inflate the NE burden. Finally, there is the possibility that a proportion of climbers who incurred injury did not go to the ED for care, but sought care at other locations such as urgent walk-in clinics or community medical facilities. The omission of these injuries could underestimate the burden of non-fatal injuries like sprains/strains or specific types of fractures.

Despite these limitations, this study provides a framework with a large sample size across the age spectrum in relation to the general types of injuries and falls. Key evidence needed to develop effective prevention strategies include: identification of any deficits in muscle strength and power performance across climbing disciplines between sexes or age groups, determination of effects of level of experience and technical competence of the climber at the time of injury, presence of other factors such as fatigue, effect of presence of spotters on the type of injury, impact of other cross training on coordination and performance, and specific environmental conditions that are related to injury in indoor and outdoor environments.

### 4.2 Conclusion

Females and climbers aged <18 years more often experience falls in indoor/rock wall environments with short fall heights, whereas more males and climbers aged 18–50 years are injured outdoors from moderate heights. Preventative efforts to address these specific injury types, and fall-related injury risk with clear understanding of the mechanisms involved, for the general climbing population in the U.S. are warranted.

## Data Availability

The original contributions presented in the study are included in the article/Supplementary Material, further inquiries can be directed to the corresponding author.
